# Loneliness and Mild Cognitive Impairment in Older Adults: A Cross‐Sectional Study in Ghana

**DOI:** 10.1002/alz70860_096487

**Published:** 2025-12-23

**Authors:** Daniel Naawenkangua Abukuri

**Affiliations:** ^1^ University of Ghana, Accra, Greater Accra, Ghana

## Abstract

**Background:**

Loneliness is an emerging public health concern in low‐ and middle‐income countries (LMICs), driven by urbanization and shifting social dynamics. Evidence suggests loneliness may increase the risk of mild cognitive impairment (MCI), a precursor to dementia. However, limited research exists on this association in LMICs, particularly in Ghana. This study investigates the relationship between loneliness and MCI among older adults in Ghana, aiming to inform dementia prevention strategies.

**Method:**

This cross‐sectional study will be conducted in urban and rural Ghana, involving 300 participants aged 60 and above. Cognitive function will be assessed using the Montreal Cognitive Assessment (MoCA), and loneliness will be measured using the UCLA Loneliness Scale. Data on sociodemographic factors (e.g., age, gender, living arrangements) will be collected. Multiple regression analysis will be used to examine the association between loneliness and MCI, controlling for confounders like physical health and socioeconomic status.

**Result:**

Preliminary findings reveal a significant link between loneliness and mild cognitive impairment (MCI). Of 300 participants, 45% (*n* = 135) reported moderate to severe loneliness, and 38% (*n* = 114) were diagnosed with MCI, with 55 participants experiencing both conditions. Those with moderate to severe loneliness had nearly double the risk of cognitive decline compared to less lonely individuals (Odds Ratio: 1.96, 95% CI: 1.4–2.7, *p* < 0.05). Further analysis showed that 80 participants reported loneliness without MCI, while 59 had MCI but low loneliness, and 106 neither reported loneliness nor MCI. Physical health and social support influenced cognitive outcomes but did not weaken the loneliness‐MCI association (adjusted β coefficient = 0.62, *p* = 0.03). Qualitative data supported these results, highlighting memory lapses, reduced focus, and diminished problem‐solving skills among lonely participants.

**Conclusion:**

Loneliness is a key risk factor for MCI in Ghana, independent of other variables. Addressing loneliness through community‐based interventions may help prevent cognitive decline and reduce dementia risk. Further research is needed to confirm these findings and explore underlying mechanisms.

